# Analysis of the Intratumoral Adaptive Immune Response in Well Differentiated and Dedifferentiated Retroperitoneal Liposarcoma

**DOI:** 10.1155/2015/547460

**Published:** 2015-01-29

**Authors:** William W. Tseng, Shruti Malu, Minying Zhang, Jieqing Chen, Geok Choo Sim, Wei Wei, Davis Ingram, Neeta Somaiah, Dina C. Lev, Raphael E. Pollock, Gregory Lizée, Laszlo Radvanyi, Patrick Hwu

**Affiliations:** ^1^Department of Surgery, Section of Surgical Oncology, University of Southern California, Los Angeles, CA 90033, USA; ^2^Hoag Memorial Hospital Presbyterian, Newport Beach, CA 92663, USA; ^3^Department of Melanoma Medical Oncology, The University of Texas MD Anderson Cancer Center, Houston, TX 77030, USA; ^4^Department of Biostatistics, The University of Texas MD Anderson Cancer Center, Houston, TX 77030, USA; ^5^Department of Cancer Biology, The University of Texas MD Anderson Cancer Center, Houston, TX 77030, USA; ^6^Department of Sarcoma Medical Oncology, The University of Texas MD Anderson Cancer Center, Houston, TX 77030, USA; ^7^Division of Surgical Oncology, The James Comprehensive Cancer Center, Ohio State University Medical Center, Columbus, OH 43210, USA; ^8^Lion Biotechnologies, Woodland Hills, CA 91637, USA; ^9^Department of Immunology, H. Lee Moffitt Cancer Center, Tampa, FL 33612, USA

## Abstract

Treatment options are limited in well differentiated (WD) and dedifferentiated (DD) retroperitoneal liposarcoma. We sought to study the intratumoral adaptive immune response and explore the potential feasibility of immunotherapy in this disease. Tumor-infiltrating lymphocytes (TILs) were isolated from fresh surgical specimens and analyzed by flow cytometry for surface marker expression. Previously reported immune cell aggregates known as tertiary lymphoid structures (TLS) were further characterized by immunohistochemistry. In all fresh tumors, TILs were found. The majority of TILs were CD4 T cells; however cytotoxic CD8 T cells were also seen (average: 20% of CD3 T cells). Among CD8 T cells, 65% expressed the immune checkpoint molecule PD-1. Intratumoral TLS may be sites of antigen presentation as DC-LAMP positive, mature dendritic cells were found juxtaposed next to CD4 T cells. Clinicopathologic correlation, however, demonstrated that presence of TLS was associated with worse recurrence-free survival in WD disease and worse overall survival in DD disease. Our data suggest that an adaptive immune response is present in WD/DD retroperitoneal liposarcoma but may be hindered by TLS, among other possible microenvironmental factors; further investigation is needed. Immunotherapy, including immune checkpoint blockade, should be evaluated as a treatment option in this disease.

## 1. Introduction

Although the majority of soft tissue sarcomas occur in the upper and lower extremities, approximately 20% are found in the retroperitoneum, where tumors can often cause significant morbidity and mortality [1]. Well differentiated (WD) and dedifferentiated (DD) liposarcoma are malignancies of adipocytic origin and the most common histologic subtype encountered in the retroperitoneum. WD tumors consist of mostly atypical adipocytes, whereas DD tumors have an additional, high grade, cellular portion [2, 3]. DD liposarcoma may arise from WD liposarcoma; however the precise relationship is still unproven.

In WD/DD retroperitoneal liposarcoma, surgery is the mainstay of treatment; however as tumors are typically massive in size (mean = 30 cm) and can invade adjacent visceral organs and critical structures, resection is often quite challenging [4, 5]. Locoregional recurrence occurs frequently and patients are subjected to multiple surgeries with the potential for increased complication rates [4]. Apart from surgery, few other effective treatment options exist. The role of radiation therapy is not well established [5]. Doxorubicin-based, cytotoxic chemotherapy is frequently given, especially for DD disease; however a recent, large retrospective analysis reported an objective response rate of only 12% [6].

In the past decade, significant advances have been made in the understanding of the molecular biology of WD/DD liposarcoma. The hallmark genetic change in this disease appears to be chromosomal amplification at 12q13–15 [2, 3, 5]. This region includes several hundred to thousands of genes, including MDM2 and CDK4. Amplification of MDM2 occurs in almost all tumors and detection by fluorescence in situ hybridization is often used in the diagnosis of WD/DD liposarcoma [2, 3]. Several novel therapies, driven by disease biology, have recently emerged and are currently being evaluated in early phase clinical trials [7]. Preliminary published data with small series of patients suggests that disease stabilization can be achieved; however, objective response rates are still dismally low: 5% for the MDM2 inhibitor, RG7112, and 3% for the CDK4/6 inhibitor, PD-0332991 [8, 9].

In melanoma, immune checkpoint blockade is an immunotherapeutic strategy that has recently been shown to have impressive objective response rates and even prolongation of survival, despite advanced stage of disease and heavy tumor burden [10–13]. These therapies inhibit the molecular checkpoints (CTLA4, PD-1) or “brakes” that arise naturally in an activated T cell. Unlike vaccines, immune checkpoint blockade does not induce targeting of a specific tumor antigen but instead maintains activation and cytotoxic function in tumor-infiltrating T cells that are already naturally sensitized to a variety of tumor antigens. By blocking both CTLA4 and PD-1 in patients with metastatic melanoma, Wolchok et al. an objective response rate of 53% with tumor shrinkage of up to 80% in many responders [12]. The clinical efficacy of immune checkpoint blockade is also being increasingly reported for other advanced solid tumors [14, 15].

Given the limited and ineffective treatment options currently available to patients with WD and DD retroperitoneal liposarcoma, we sought to study the natural tumor microenvironment from an immunologic standpoint as the first step to explore the potential feasibility of immunotherapy in this disease. In contrast to myxoid liposarcoma which have high expression of the cancer testis-antigen NY-ESO-1 [16], to our knowledge, no consistent and reliable tumor antigen has ever been identified in WD/DD liposarcoma, making vaccine strategies less appealing. Our aim was to study the adaptive immune response and specifically the tumor-infiltrating T cells and their expression of PD-1. This data can then be used to guide further evaluation of immune checkpoint blockade strategies in WD/DD liposarcoma.

## 2. Material and Methods

Approval for all portions of this study was obtained by the Institutional Review Board at The University of Texas, MD Anderson Cancer Center.

### 2.1. Fresh Tumor Processing and Analysis by Flow Cytometry

Fresh tumor resected at surgery was closely examined in pathology and nonnecrotic, more fibrous/less fatty portions of tumor were excised and brought to the laboratory for study. In a sterile tissue culture hood, tumor tissue was further dissected to remove visible blood vessels and areas of hypervascularity. Tissue processing techniques used for isolation of tumor-infiltrating lymphocytes (TILs) in melanoma [17, 18] were applied and optimized for liposarcoma. In brief, tumor tissues were placed in serum-free RPMI medium containing supplemental antibiotics and kept at 4°C until ready for use. Tumor chunks were processed by first sharply dicing tissue into smaller, 3-4 mm pieces followed by 2-3 h of enzymatic digestion at 37°C in a rocker, using a cocktail containing collagenase (3%), hyaluronidase (75 *μ*g/mL), and DNAse (250 U/mL). The resulting cell suspension was then washed in PBS and pipetted across a 70 micron filter to remove debris. Ficoll density centrifugation (75%/100%) was then used to remove tumor cells and erythrocytes, enriching for immune cells. Fluorescently labeled antibodies against the cell surface markers CD3, CD56, CD19, CD4, CD8, PD-1, and 4-1BB (all from BD Biosciences, San Jose, CA) were incubated with immune cells, along with a viability marker (Live/Dead Fixable Aqua stain, Invitrogen, Life Technologies, Grand Island, NY). Surface marker expression on stained cells was determined using a multicolor FACS Canto II flow cytometer and the data was analyzed using FlowJo software.

### 2.2. Immunohistochemistry of FFPE Tissue

Formalin fixed paraformaldehyde embedded (FFPE) tumor tissue was obtained from our institutional pathology archives and 4-micrometer sections were cut. Tissue sections were deparaffinized in xylene and rehydrated through graded alcohols (100%, 95% to 80%). Antigen retrieval was carried out for 30 minutes in citric acid buffer (pH 6.0). After cooling down, the slides were thoroughly washed in distilled water and washed 3 times in 1xPBS, 2 minutes each. Endogenous peroxidase activity was quenched by immersion in 3% hydrogen peroxide (Sigma) in methanol for 10 minutes at room temperature followed by rinsing for 2 minutes in 1xPBS 3 times. Sections were then incubated with primary anti-DC-LAMP mouse antibody (clone 104G4, 1 : 100, Imgenex, San Diego, CA) for 30 minutes according to the manufacturers' instructions (Polink TS-MMR-Hu A Kit, GBI Labs, Bothell, WA). Visualization was performed with the DAB substrate supplied in the kit. Then mixed primary anti-CD4 mouse antibody (clone 4B12, 1 : 40, Leica Microsystems, Buffalo Grove, IL) and anti-CD8 rabbit antibody (clone EP1150Y, 1 : 200, Abcam, Boston, MA) were incubated and visualization was performed with AP-red for CD4 and emerald chromogen (green) for CD8 supplied in the kit. The slides were counterstained with hematoxylin and cover slipped with PerMount. For positive controls, sections of human tonsil tissues were used. Omission of the primary antibodies for tonsil tissue was used as negative controls for staining. Positive cells showed a brown, red, or green intense staining, while negative controls and unstained cells were blue.

### 2.3. Clinicopathologic Correlation and Statistical Methods

Clinical outcome data was obtained from a retrospective institutional sarcoma database for patients with and without TLS identified in available FFPE tumor sections. Kaplan-Meier curves were used to estimate recurrence-free and overall survival (RFS, OS) between patient groups. Comparisons of RFS and OS between patient groups were carried out using log-rank tests. All tests were two-sided and *P* values <0.05 were considered statistically significant. Statistical analysis was carried out using SAS version 9.3 (SAS Institute, Cary, NC). Statistical plotting was performed using Spotfire S+ 8.2 (TIBCO Inc., Palo Alto, CA).

## 3. Results

### 3.1. Tumor-Infiltrating Lymphocytes in Fresh Tissue

Tumor-infiltrating lymphocytes (TILs) were isolated from all resected retroperitoneal liposarcoma specimens (*n* = 8) included in the study (Table 1). TILs were identified independent of histology (WD versus DD), disease status (primary versus recurrent), or receipt of chemotherapy or radiation therapy prior to resection.

**Figure 1:**
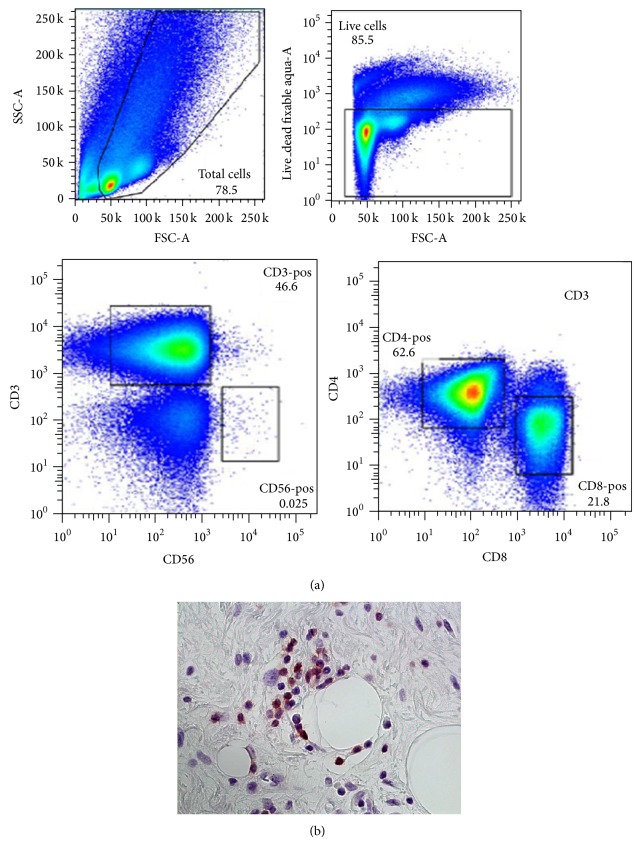
Tumor-infiltrating lymphocytes or TILs in WD/DD retroperitoneal liposarcoma. (a) Representative analysis by flow cytometry with gating schema for identification of CD3, CD4, and CD8 T cells. (b) Immunohistochemistry demonstrating intratumoral presence of CD8 T cells (brown), 400x magnification.

TILs consisted of a substantial population of CD3 T cells (Figure 1) and by flow cytometric analysis, the majority of these cells were CD4 “helper” T cells with a CD4 to CD8 ratio of 4.2 (range 2.0–8.6) (Table 1). CD8 “cytotoxic” T cells, however, were found in all tumors and represented an average of 20% (8–31) of the total CD3 T cell population. CD19 B cells and CD56 NK cells were seen in some tumors, generally with a low frequency (data not shown). Presence of select immune populations, including CD8 T cells, was verified by immunohistochemistry (Figure 1(b)).

**Figure 2:**
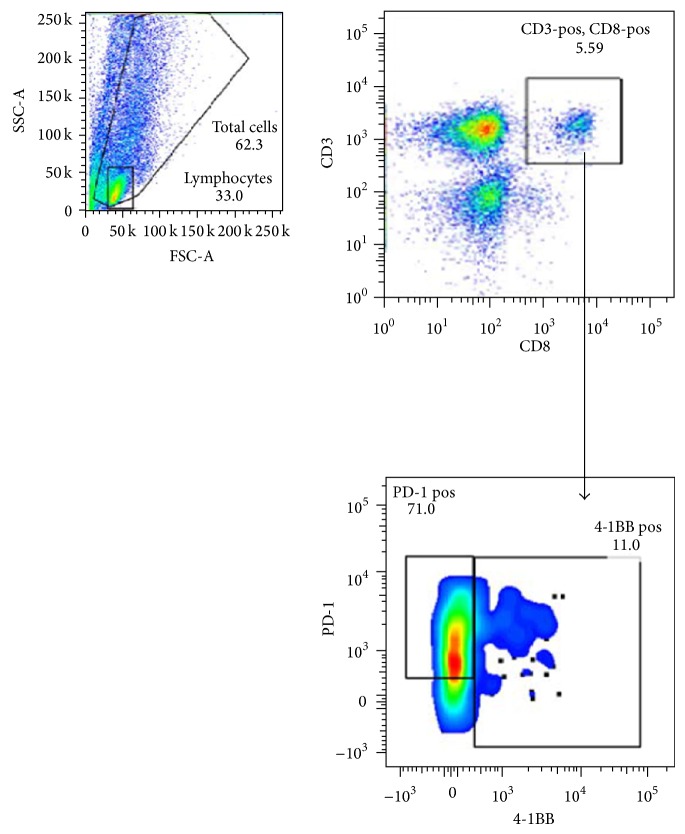
Expression of PD-1 and 4-1BB among the TIL CD8 population.

Further analysis of surface marker expression on the cytotoxic CD8 T cell population demonstrated a high frequency of expression of the immune checkpoint molecule, PD-1, which was seen in 65% (57–73) of cells (Table 2, Figure 2). In contrast, there was a low frequency of CD8 T cells with expression of the costimulatory molecule, 4-1BB, seen in 10% (3–19) of cells (Table 2, Figure 2).

### 3.2. Tertiary Lymphoid Structures in FFPE and Clinical Correlation

**Figure 3:**
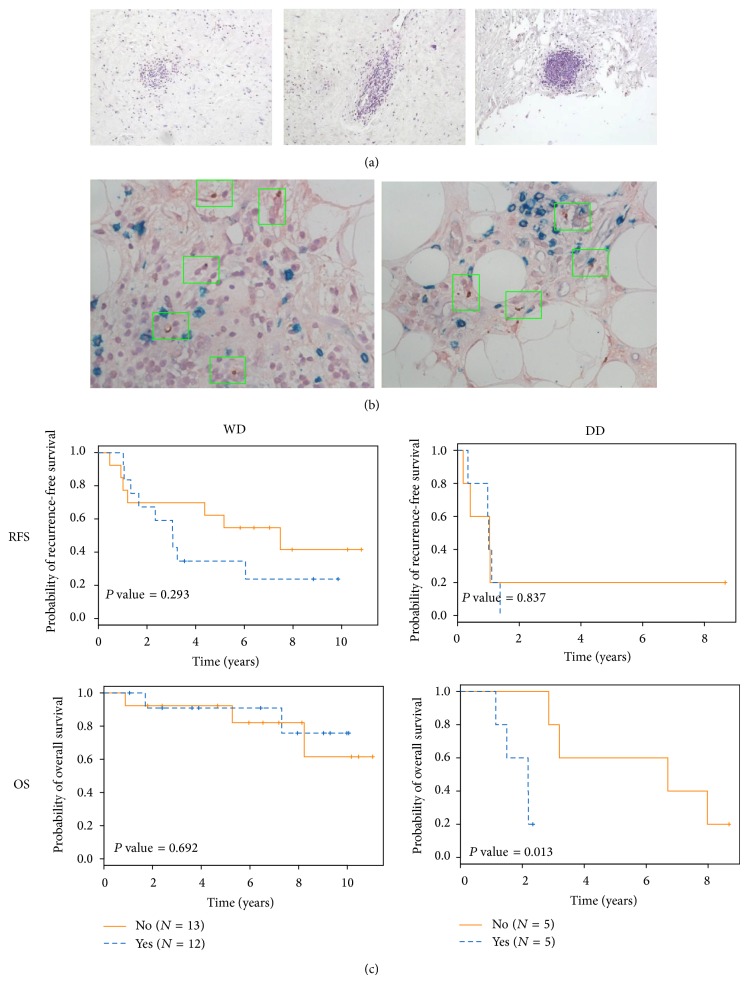
Intratumoral tertiary lymphoid structures (TLS) in WD/DD retroperitoneal liposarcoma. (a) General histologic appearance of TLS with varying levels of complexity and size, 100x magnification. (b) Immunohistochemistry for DC-LAMP (brown dots), a marker for mature dendritic cells, CD4 (red) and CD8 (green). Green boxes denote areas with DC-LAMP positive cells. 400x magnification. (c) Clinical outcome for patients with and without intratumoral TLS with recurrence-free survival (RFS) and overall survival (OS) shown.

Tissue sections from archived, FFPE tumor (*n* = 35) were analyzed by H&E and/or immunohistochemistry for presence of intratumoral tertiary lymphoid structures or TLS as described in WD liposarcoma, previously [19]. TLS were generally found in perivascular locations; however TLS could also be found in adipocytic areas of tumor. Varying levels of “architectural maturity” of TLS were observed, ranging from simple aggregates of immune cells to more complex structures resembling germinal centers, typically found within lymph nodes (Figure 3(a)). Occasionally, even macroscopic intratumoral TLS were seen (data not shown). No consistent differences in TLS characteristics (intratumoral location, maturity/size) were noted between WD and DD tumors. By immunohistochemistry, mature dendritic cells expressing DC-LAMP were identified within TLS (Figure 3(b)). Costaining for CD4 and CD8 demonstrated apparent juxtaposition of these mature dendritic cells next to CD4 T cells, suggestive of classic antigen presentation.

In total, TLS were identified in available tissue sections in 12 out of 25 (48%) of WD and 5 out of 10 (50%) of DD retroperitoneal liposarcoma tumors. Presence of TLS was associated with worse recurrence-free survival in patients with WD liposarcoma and worse overall survival in those with DD liposarcoma (Figure 3(c)). No differences in disease status (primary versus recurrent) or prior treatment (chemotherapy, radiation therapy) were noted between patients with and without intratumoral TLS for either histology.

## 4. Discussion and Conclusions

The first suggestion of an active immune component to WD/DD liposarcoma, at least in some tumors, was made in the late 1990s by two independent descriptions of an “inflammatory” variant of WD liposarcoma [20, 21]. Our own group reported a more contemporary characterization based on immunohistochemistry, which revealed the potential for a naturally occurring adaptive immune response [19]. In the current study, we expanded on our previous work to include all “noninflammatory” WD and DD retroperitoneal liposarcoma and also used flow cytometry to provide deeper analysis of the immune cells. We focused on T cells, a critical component of the adaptive immune response, found in the tumor microenvironment.

Tumor tissue obtained from surgery was used for study as this has direct relevance to patients and importantly, in WD/DD liposarcoma, there are no validated immunocompetent animal models available. The vast majority of preclinical models are xenografts established in immunodeficient mice [22]. Even in these xenograft models, in vivo growth is not consistent and in fact, tumor uptake is largely limited to the higher grade, DD tumors (personal communication, D. Lev). One exception is a report of a genetically engineered mouse model, in which spontaneous WD liposarcoma serendipitously developed in IL-22 overexpressing mice subjected to a high fat diet [23]. Tumors were shown to have MDM2 amplification confirming the diagnosis; however no further independent validation of this model has been done, to our knowledge. Interestingly, a prominent immune infiltrate was seen in tumors found in these mice, although further characterization was not reported.

The data from the current study confirms the presence of a naturally occurring, adaptive immune response within liposarcoma tumors, including presence of cytotoxic CD8 T cells (Figure 1). The high frequency of PD-1 expression and low 4-1BB expression (Figure 2) imply that these tumor-infiltrating CD8 T cells have been sensitized to tumor antigen but are no longer activated [24]. Interestingly, no clear differences in the frequency of CD8 T cells or the expression of PD-1/4-1BB were seen when comparing histology (WD versus DD, higher grade) or disease status (primary versus recurrent) (Tables 1 and 2). In separate experiments, we were able to expand these tumor-infiltrating lymphocytes or TILs in vitro, from 300 to almost 2000-fold using IL-2 and standard methods established for melanoma (data not shown). Taken together, our data suggests that in WD/DD retroperitoneal liposarcoma, the T cells can traffic to the tumor microenvironment and have the capacity to proliferate but lack effective antitumor function, likely from deactivation.

Although we did not directly analyze PD-1 expression by immunohistochemistry, the presence of this marker on immune cells within WD/DD liposarcoma tumors has been confirmed in a published report [25] and recently in an abstract presentation [Pollack et al., CTOS 2014]. Both studies looked at immunohistochemical expression of PD-1 in a variety of soft tissue sarcomas, which included a small cohort of liposarcoma cases. Our study is the first, to our knowledge, to demonstrate expression of this marker by flow cytometric analysis.

The etiology for cytotoxic CD8 T cell deactivation is unknown and likely multifactorial. The majority of the T cell population found in liposarcoma tumors are actually CD4 “helper” T cells. Intracellular staining with FoxP3 was positive in only a few, isolated cells (data not shown) suggesting that immunosuppressive, regulatory T cells are actually rare in the tumor microenvironment for WD/DD retroperitoneal liposarcoma. Other immunosuppressive cell types including myeloid derived suppressor cells or MDSCs and tumor-associated macrophages, however, likely also exist in the tumor microenvironment and are currently being investigated. A variety of tumor-derived factors both soluble (e.g., cytokines – IL-10, TGF-beta) and on the cell surface (e.g., PD-L1) may also lead to deactivation of CD8 T cells and remain to be defined.

Tertiary lymphoid structures or TLS may further hinder the antitumor response in WD/DD retroperitoneal liposarcoma. TLS have been described in non-small cell lung cancer, colorectal cancer, and melanoma and are likely intratumoral sites of antigen presentation or “ectopic” lymph nodes [26–28]. In liposarcoma, this concept is also supported by our observation of DC-LAMP positive, mature dendritic cells juxtaposed next to CD4 T cells (Figure 3(b)). In contrast to published reports in other solid tumors, our preliminary data suggest that, in liposarcoma, TLS may possibly be associated with worse clinical outcome. This data is limited by the relatively small number of cases studied and may be affected by sampling error with the sections of tumor that were available to us for TLS analysis. Nonetheless, our findings lead to the hypothesis that antigen presentation may be different on a cellular or cytokine level in liposarcoma versus other solid tumors. We observed varying levels of “architectural maturity” with TLS in liposarcoma (Figure 3(a)); given the typically large size of these tumors, perhaps TLS have evolved from antitumor to more protumor during the course of tumor growth. Alternatively, as liposarcomas have very few and inconsistent mutations in contrast to melanoma or lung cancer [29, 30], TLS in liposarcoma may be sites of antigen presentation against nonmutated antigens for which tolerance mechanisms are likely to exist. Finally, WD/DD liposarcoma does not disseminate to regional lymph nodes and having intratumoral TLS as potentially the only site antigen presentation may somehow negatively affect the antitumor immune response. Further studies with larger sample sizes are needed to validate our findings and explore these hypotheses.

From a treatment standpoint, our findings provide strong rationale to further evaluate the therapeutic potential of immunotherapy in WD/DD retroperitoneal liposarcoma. Immune checkpoint blockade (e.g., anti-CTLA4 or anti-PD-1) is particularly attractive as this can reactivate cytotoxic CD8 T cells, already sensitized to tumor antigen. The existence of an infiltrate of these immune cells within tumors puts WD/DD liposarcoma at an advantage in terms of potential for response to immune checkpoint blockade [31]. One potential biomarker to predict treatment response is PD-L1, the ligand for PD-1, found on tumor cells and antigen presenting cells [15]. In the current study, we did not analyze PD-L1 expression; however this data has been recently reported in soft tissue sarcoma [25]. Among the liposarcoma cases, 2 out of 4 (50%) WD and 2 out of 3 (67%) DD tumors expressed PD-L1 by immunohistochemistry. Other investigators have presented data in liposarcoma, thus far only in abstract form, showing the full spectrum of tumor PD-L1 expression from zero [D'Angelo et al., ASCO 2014] to 100% [Movva et al., ASCO 2014]. This wide variation is consistent with a previous report in melanoma which has suggested that PD-L1 expression fluctuates in relation to inflammation and other factors within the tumor microenvironment [32]. Other biomarkers for tumor response to immunotherapy are currently being explored.

**Figure 4:**
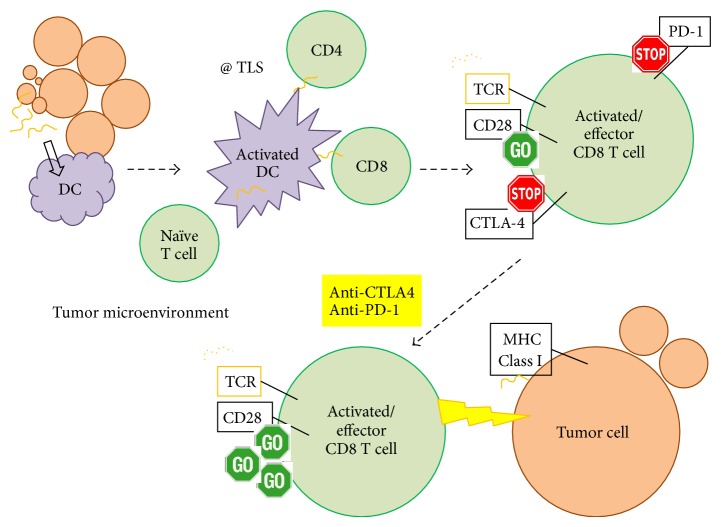
Summary of the intratumoral adaptive immune response in WD/DD retroperitoneal liposarcoma and the potential clinical utility of immune checkpoint blockade. Brown circles = tumor cell, yellow squiggle = tumor antigen, purple shapes = dendritic cell (DC), and green circles = T cell; TLS = tertiary lymphoid structure; TCR = T cell receptor; MHC = major histocompatibility complex.

We have summarized our findings in a schematic shown in Figure 4. Adaptive immune responses have been identified in other soft tissue sarcomas [33, 34]. Immunotherapy has the potential for efficacy in soft tissue sarcoma but the challenge will be to identify an appropriate strategy for each histologic subtype based on preclinical and translational data. Our results provide the initial framework to guide more detailed immunologic study in WD/DD retroperitoneal liposarcoma, which is currently ongoing in our laboratory. Given the lack of effective treatment options, immunotherapy and, in particular, immune checkpoint blockade should be further evaluated as it may offer new hope for patients with this disease.

## Figures and Tables

**Table 1:** Tumor-infiltrating lymphocytes or TILs in WD/DD retroperitoneal liposarcoma.

Case	Histology	Disease status	Preop chemo	Preop Rad. Tx	Tumor size (g)	%CD4/%CD8
1	WD	R	N	N	12	61/31
2	WD	P	N	N	13	64/26
3	WD	R	N	N	2	71/28
4	WD	R	N	N	11	69/8
5	WD	R	N	N	2	57/12
6	DD	R	Y	N	6	63/22
7	DD	R	N	N	6	72/11
8	DD	P	Y	N	6	69/19

**Table 2:** Expression of PD-1 and 4-1BB among the TIL CD8 population.

Histology	Disease status	% PD-1 (of CD8)	% 4-1BB (of CD8)
WD	P	73	3
DD	R	71	11
WD	R	61	11
WD	R	61	19
WD	P	57	5
